# A Person-Centered Analysis of Meaning in Life, Purpose Orientations, and Attitudes toward Life among Chinese Youth

**DOI:** 10.3390/bs13090748

**Published:** 2023-09-07

**Authors:** Hong Wang, Xiaosong Gai, Songliang Li

**Affiliations:** 1 School of Psychology, Northeast Normal University, 5268 Renmin Street, Changchun 130024, China; wangh011@nenu.edu.cn (H.W.); songlian@asu.edu (S.L.); 2 Research Center of Mental Health Education in Northeast Normal University, Key Research Institute of Humanities and Social Science in Universities in Jilin Province, Changchun 130024, China

**Keywords:** meaning in life, purpose orientations, attitudes toward life, youth

## Abstract

**Background**: Meaning in life, purpose orientations, and attitudes toward life have a significant impact on youths’ well-being. The purpose of this study is to investigate the developmental trends of youths’ meaning in life, purpose orientations, and attitudes toward life. **Methods**: The sample consisted of 94,219 students aged 13 to 23 years (M = 16.67, SD = 2.70). Person-centered analysis, MANOVA, and an independent sample *t*-test were used to analyze the data. **Results**: Most youths were in the “search” or “presence” type in terms of meaning in life status. Fewer students were identified as being in the “ruminative exploration” or “diffusion” type. Very few were in the “precontemplation” or “foreclosure” stages. The status of the sense of meaning did not change significantly with age. Second, in terms of purpose orientations, Chinese youths consider family well-being and personal growth to be the most important goals, whereas personal well-being and social promotion are less important. Third, in terms of attitudes toward life, most young people take an active, accepting, and optimistic view of their lives, seeing life as an experience or process, rather than a good or bad result. Fourthly, the age of 16 was found to be a significant turning point. More emerging adults were in the “presence” state than adolescents, but their attitudes toward life were not as positive as those of adolescents. **Conclusions**: This study reveals that Chinese youth consider the question of meaning in life as early as age 13. Most of them were in the state of “searching for meaning”. Therefore, education about meaning in life should be integrated into the primary school context. Family well-being is emphasized by Chinese youth because of the collectivist culture. Family well-being and personal growth should be recognized, and social promotion should be enhanced in guidance of Chinese youth’s meaning acquisition.

## 1. Introduction

Pursuing meaning in life and purpose orientations is a crucial developmental task during youth [1,2,3,4]. Scholars have emphasized the need to consider life concerns during adolescence and emerging adulthood. Developing a purpose in life, meaning in life, and attitudes toward life that will shape one’s future is critical during youth [2] and increasingly important throughout life [3]. However, classificatory, stage, or status models, all of which share similarities in measuring meaning in life, purpose in life, and attitudes toward life, present challenges that have long been recognized in developmental psychology. As a result, meaning in life, purpose in life, and attitudes toward life are likely to undergo some developmental growth between adolescence and early adulthood. In this period, adolescence and emerging adulthood are transitional periods where individuals may first begin to consider abstract concepts such as purpose, meaning in life, and attitudes toward life [2,3]. Erikson proposed that the primary psychosocial task during youth is identity formation, which provides a profound sense of ideological commitment and enables individuals to understand their place in the world [1]. On the other hand, failure to develop a sense of identity can lead to role confusion, causing youth to question their essential personality traits, their self-concept, and how others perceive them. At once, youth may doubt the meaning and purpose of their existence, leading to feelings of loss and depression [5]. Thus, in this stage of life youth are expected to examine their meaning in life, purpose orientations, and attitudes toward life.

### 1.1. Meaning in Life

Meaning in life refers to the extent to which individuals grasp, interpret, or find significance in their existence and their perception of having a purpose, mission, or overarching objective [6]. Developing meaning in life during adolescence involves exploring and cultivating a sense of purpose while identifying one’s developmental stage [7]. According to Steger, meaning in life consists of the search for meaning and the presence of meaning, which affects adolescents differently [6].

Research has shown that the presence of meaning is more conducive to positive adolescent development [8]. Moreover, the presence of meaning is positively associated with general well-being [9]. The presence of meaning is also associated with health behaviors such as sleep quality [10]. Conversely, the search for meaning is positively associated with depression, anxiety, emotional distress, suicidal ideation, and lower psychological well-being [9,11]. For instance, in a study of 18-year-olds, those who perceived life as meaningless were more likely to experience anxiety, have lower levels of physical health, and have higher intentions to seek meaning than those who perceived life as meaningful [12].

Furthermore, it is important to recognize that adolescents’ perceptions and thoughts about meaning in life are not limited to the search for meaning and the presence of meaning [12]. Research has consistently shown a strong relationship between self-identity development and meaning in life, suggesting that the latter should follow a similar developmental structure [13]. However, when considering the question of meaning in life within a developmental model, there may be more types. First, according to the stages of behavioral change in the transtheoretical model, there may be a “precontemplation” stage in which one does not begin to think about meaning in life; second, according to Marcia’s theory of self-identity, there may be a state of “diffusion” in which one seeks but fails to establish and a state of “foreclosure” in which one is dominated by adult concepts before exploring autonomously [14]. Third, some adolescents may be in a state of “ruminative exploration” in which they do not know what to do until they know what they want to do.

Some researchers have qualitatively used cluster analysis to investigate the division of meaning based on Marcia’s findings [13,14,15,16,17]. It is important to note that cluster analysis can help identify discrete subgroups of people with different levels of public engagement within a given sample. However, this method is dependent on the data set being examined [17]. Therefore, a comprehensive approach that includes both qualitative and quantitative research may be more appropriate for measuring meaning in life [17].

On this basis, this study adopts the Sense of Meaning in Life Scale, which proposes six developmental stages of meaning formation in adolescents [18]. Liu proposed precontemplation, foreclosure, search, ruminative exploration, diffusion, and presence as developmental stages of meaning in life formation in Chinese adolescents [18]. Meanwhile, this study uses a quantitative method to provide a framework for understanding youths’ meaning in life, and an attempt is made to determine the percentage of secondary school students in each state of meaning in life and their age trends.

### 1.2. Purpose Orientations

Purpose in life can be defined as having a personally meaningful goal that drives active participation in life and provides a sense of direction in one’s existence [19]. Furthermore, previous research on purpose in life has focused on a general sense of purpose without delving into the specifics of an individual’s purpose [3,4]. However, this approach has been criticized for assuming that any sense of purpose is beneficial regardless of its content [3]. Recent research on purpose content highlighted the importance of assessing its quantity and quality since different purpose contents can have distinct effects on an individual’s well-being [3]. As a result, there have been calls for greater emphasis on purpose content and not just subjective feelings of purpose [3,4]. Hence, scholars have shifted their attention to investigating the actual content of an individual’s purpose in life.

Nevertheless, purpose content can be classified as having purpose orientations, which refer to the specific goals individuals aim to achieve [20]. Understanding the nature of one’s purpose can guide purpose development and determine the relevance of different pursuits [21]. Pursuing different life purposes can have varying effects on individuals. For example, external goals such as wealth and physical appearance are associated with lower levels of happiness and higher levels of distress than internal goals such as relationships and health [22]. Prosocial purposes, such as contributing to society, have been associated with higher life satisfaction [23], greater happiness [24], and better academic performance [25]. In addition, the pursuit of socially oriented purposes can promote positive adolescent development [26], whereas self-oriented purposes are associated with lower levels of happiness [24].

Meanwhile, as scholars aim to understand the impact of environmental trends on the developmental challenges faced by today’s youth, there has been a growing interest in understanding purpose in life across different cultures [27]. People create their purpose in life by drawing on their characteristics, life experiences, and cultural beliefs and norms, which serve as the basis for creating purpose. The values of purpose establish the needs of civilization. Personal success is more highly valued as an individual value in industrialized civilizations, whereas family loyalty is more highly valued in traditional communities [28]. Researchers have emphasized the need to study the concept of purpose in life in non-American and non-individualistic cultures in order to understand how purpose is perceived in environments that emphasize connectedness [17,28]. This emphasis makes the present study of a Chinese sample unique. Collectivism and Confucianism have profoundly influenced the shared cultural experiences of adolescents of Chinese ancestry, regardless of their geographic origins across regions and societies such as Hong Kong, Singapore, and Taiwan [17]. In contrast to Western societies that promote self-actualization as a life goal, Chinese culture places a strong emphasis on the collective happiness of individuals [29]. Ren, a collective virtue that includes attributes such as kindness, selflessness, and diligence, is the highest aspiration and center of purpose in life according to Confucianism (Confucius, ca. 479 BC–221 BC). Ren is seen as a personal virtue with the ultimate goal of contributing to a peaceful society [30]. Furthermore, purpose in life has received considerable support and analysis in societies that prioritize individualism [31]. However, how might the contextual elements surrounding the development of purpose differ (or be similar) among Chinese adolescents in collectivist or interdependent societies, where harmony in relationships is vital and self-sufficiency and personal ambition are not as emphasized? For instance, collectivist values may encourage youth to expand their purpose in life beyond themselves and promote purposes sanctioned by their families [32].

The purpose orientations in this study are classified into four types: societal promotion, personal growth, family well-being, and personal well-being [33]. The study uses the self-statement and forced-choice scales of purpose orientations to better understand the types of goals that Chinese youth value and do not value. Therefore, this research contributes to a more comprehensive understanding of Chinese adolescents’ purpose orientations.

### 1.3. Attitudes toward Life

Meaning in life formation and purpose attitudes can be related to how youth pursue life or their attitudes toward life. Individuals’ disposition and behavior towards people, objects, and situations related to existence are referred to as attitudes toward life [34]. It encompasses both the cognitive and emotional aspects of life and can be positive or negative [34]. Positive attitudes are associated with higher levels of happiness, life satisfaction [5,35], and physical health [36], whereas negative attitudes can lead to academic anxiety and poor interpersonal communication [37]. Therefore, it is essential to understand youths’ current attitudes toward life to address any negative attitudes.

On the other hand, some researchers often confuse meaning in life, purpose orientations, and attitudes toward life. For instance, the Life Attitude Profile (LAP), developed by Reker and Peacock, assesses individuals’ motivation to discover meaning and purpose [38]. Similarly, Ho’s Life Attitude Profile explores people’s desire for meaning and purpose [39]. More recently, Hsieh and Pan developed the Attitude Toward Life Scale (LAS) based on Ho’s work, which measures college students’ positive and negative attitudes toward life [34,39]. In contrast to the aforementioned measurement tools, the present study aims to distinguish attitudes toward life from the general presence of meaning in life and purpose in life. Therefore, the authors adopt Chen et al.’s characterization of attitudes toward life as acceptance vs. excessive demands, active vs. passive, process vs. result, and optimism vs. pessimism [40].

### 1.4. Youths’ Meaning in Life, Purpose Orientations, and Attitudes toward Life Are Related to Age

Despite previous research, there is little evidence that youths change their meaning in life, purpose orientations, and attitudes toward life as they age. Therefore, it is crucial to have the necessary knowledge to capture the formation or development of these concepts during youth. Whereas adults are more likely than youth to experience the formation of meaning in life, defined purpose orientations, and defined attitudes toward life, little research has focused on youth at different ages [41]. Scholars have emphasized the need to consider life concerns during adolescence and early adulthood. However, there is limited evidence on how young people develop and evolve at different ages.

Nevertheless, existing evidence suggests that the ninth and seventh grades are typically regarded as the most challenging and exhausting stages of a student’s educational career in China [5]. This is mainly due to the demanding requirements of the Comprehensive Assessment Program for junior high school students and the General Scholastic Ability Test and Advanced Subjects Test for senior high school students, all of which are required for admission to senior high school or college. Students must prioritize their academic performance at all stages of their education to prepare effectively for standardized tests. This can lead to persistent stress and negative emotions that can affect their daily experiences [42]. In contrast, a study of secondary school students in the United Kingdom found that exams and assessments were not threatening or harmful to students’ psychological well-being. They found no significant difference in depression between 9th and 11th graders [43].

### 1.5. The Present Study

Therefore, summarizing the results of previous studies, it is not difficult to highlight the following points. First, adolescence and early adulthood are the critical periods for the development of meaning in life, purpose orientations, and attitudes toward life, so it is necessary to conduct research on this group. Second, the division of the state of meaning in life in previous studies was not comprehensive and systematic enough, mainly including the search for meaning and the presence of meaning. The meaning in life scale used in this study expands meaning in life into six states, which are more comprehensive and systematic. Third, due to cultural differences, purpose orientations may be culturally specific in a non-personalistic cultural environment. Therefore, this study aims to explore the meaning in life, purpose orientation, and attitude toward life characteristics of Chinese adolescents in a non-individualistic cultural environment. Fourth, the concept of attitudes toward life has been confused with the concept of meaning in life in previous studies, and the attitude toward life scale used in this study distinguishes between them. Fifth, there is not enough clarity about the changes in meaning in life, purpose orientations, and attitudes toward life with age development, and more research is needed. Finally, this study was conducted with a large sample that is broadly representative. It is more comprehensive and systematic in describing the current status of adolescents’ meaning in life, purpose orientations, and attitudes toward life from ages 13 to 23, as well as the trend of change with age. This study achieved this goal through a cross-sectional survey. On this basis, this study answered the following four questions: (1) Are Chinese adolescents contemplating life? (2) What are the goals of Chinese adolescents? (3) Do Chinese adolescents have a positive attitude toward life? (4) How do meaning in life, purpose orientations, and attitudes toward life of Chinese youth change with age?

## 2. Materials and Methods

### 2.1. Participants

This study used a convenient sampling cross-sectional survey to collect data from 104,377 young people aged 13–23 years in 31 provinces, municipalities, and autonomous regions in mainland China. Data were collected from junior high school, high school, and college students. Both paper and electronic versions of the survey were used, and after removing invalid questionnaires, 94,219 valid questionnaires were included in the final analysis. The sample consisted of 40,883 (43.4%) male participants and 53,336 (56.6%) female participants, an adolescent sub-group (13–18 years old = 64,712), and an emerging adult sub-group (19–23 years old = 29,507), with an average age of 16.67 years (SD = 2.70). The specific sample distribution is shown in Table 1.

### 2.2. Procedure

The study recruited students from various schools to complete paper and online questionnaires after class. The widely used wjx platform (https://www.wjx.cn) was used to distribute the questionnaire. In addition to standard scales, five lie detection items were included. These items were designed to identify respondents who might not be answering truthfully, e.g., “I have never cried”, “I have never missed an appointment”, “I have never scolded others”, “I never eat snacks”, and “I have never told a lie”. Respondents who gave inconsistent answers to four of the five questions were excluded from the final sample. After completing the questionnaire, participants were able to access their results.

### 2.3. Measures

#### 2.3.1. Meaning in Life

Meaning in life was measured using the Sense of Meaning in Life Scale [18]. The scale contains 26 items on a 5-point Likert scale, ranging from 1 (completely disagree) to 5 (completely agree). The Sense of Meaning in Life Scale was created to measure six developmental stages of meaning formation in Chinese adolescents. It reflects the fact that one or more of these stages will predominate during adolescence. The six developmental stages are precontemplation, foreclosure, search, ruminative exploration, diffusion, and presence. Precontemplation is the absence of any thought or reflection about meaning in life. Foreclosure is accepting meaning in life as transmitted by parents or elders without giving it much thought or reflection. Searching involves actively exploring questions about meaning in life. Ruminative exploration is when a person has explored questions about meaning in life but has been unable to make a choice and has experienced some symptoms of maladjustment (anxiety, low self-esteem, etc.) as a result. Diffusion is when a person tries to explore questions about meaning issues but does not get answers and does not want to explore further. Presence means that a clear meaning in life has now been formed. Reliability was measured by Cronbach’s alpha, indicating good reliability for the six developmental stages: precontemplation α = 0.74, foreclosure α = 0.76, search α = 0.83, ruminative exploration α = 0.72, diffusion α = 0.78, and presence α = 0.71.

#### 2.3.2. Purpose Orientations

Purpose orientations were measured using the Youth Purpose Orientations Scale in two forms, a Likert-scale form and a forced-choice form. The Youth Purpose Orientations Scale consists of 19 items [33]. The scale was developed in a Chinese context to assess the motivation of junior high school students, high school students, and college students to pursue four different purpose orientations. Five items measured social promotion, five items measured personal growth, four items measured family well-being, and five items measured personal well-being. The reliability of the subscales of the Youth Purpose Orientations Scale was high: overall scale α = 0.93, social promotion α = 0.90, personal growth α = 0.89, family well-being α = 0.81, and personal well-being α = 0.91. The self-reported form of the scale was scored on a 5-point Likert scale ranging from 1 (not at all important) to 5 (very important). In addition, the forced-choice form was assessed using a two-choice scale. Two choices were presented for each of the 18 items. For example, for “Do you care more about your future life path?” participants could answer by prioritizing A = “The development of the country or society” or B = “Opportunities for growth and progress”. The four purpose orientation subscales were ranked according to the highest reported degree for level of importance.

#### 2.3.3. Attitudes toward Life

Attitudes toward life were assessed using the Attitudes Toward Life Scale, developed by Chen et al. [40]. The scale measures four subscales: active vs. passive, acceptance vs. excessive demands, process vs. result, and optimism vs. pessimism. The subscales are measured by 12 items. Each item has two options on a 5-point Likert scale. For example, an active vs. passive attitude toward life is perceiving life as a free choice or passively accepting it, so the Likert scale ranges from 1 = (strongly agree with perceiving life as a free choice) to 5 = (strongly agree with passively accepting life). Moreover, a persistence-acceptance attitude toward life is related to whether it is brave to face or difficult to accept suffering and setbacks. A process vs. result in attitude toward life is whether individuals are more concerned with the process and experience or the result and achievement of life. Finally, optimism vs. pessimism refers to whether individuals are optimistic or pessimistic about their future. The internal consistency of the scale was measured using Cronbach’s alpha and was found to be good, with an overall scale of α = 0.80. Confirmatory factor analysis (CFA) was performed using Mplus with model fit indices of χ^2^/df = 18.69, CFI = 0.92, TLI = 0.90, RMSEA = 0.073, and SRMR = 0.046.

### 2.4. Statistical Analysis

All statistical analyses were carried out using IBM SPSS (version 22.0) and Mplus (version 8.0) software. Each age group from 13 to 23 was treated as a group, as the sample was large enough to allow this. First, a person-centered analysis was used to calculate the distribution of meaning in life developmental stages and forced-choice purpose orientations across ages. The meaning in life dimensions were ranked from highest to lowest according to the participant’s responses. Similarly, the forced-choice purpose orientations were ranked from high to low. In the case of a tie, they were coded as “other”. Hence, there were 31 different distributions of purpose orientations, each of which differed in its ranking from highest to lowest. Moreover, to measure age differences, we first tested the measurement equivalence of the meaning in life, purpose orientations, and attitudes toward life scales. Then, a multifactor analysis of variance (MANOVA) was conducted across meaning in life, purpose orientations, and attitudes toward life. Bonferroni follow-up comparisons were used to validate the results. Finally, differences between the adolescent and emerging adult groups were analyzed using independent sample *t*-tests.

## 3. Results

### 3.1. Analysis of the Meaning in Life

**Figure 1:**
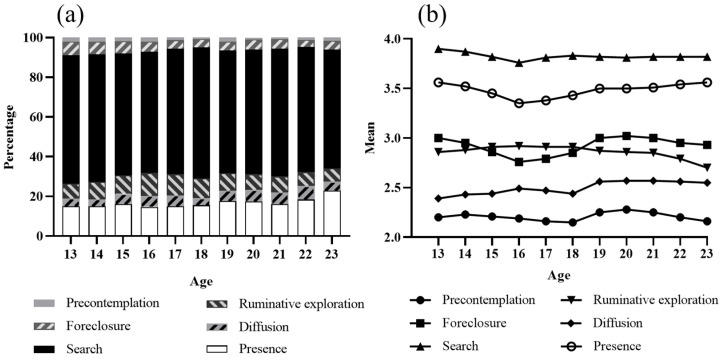
(**a**) Percentages of meaning in life developmental stages across age groups. (**b**) Means of meaning in life developmental stages across ages.

First, we set out to understand the changes in the various states of youths’ sense of meaning in life as they develop with age. Youths of different ages were analyzed using the person-centered method. The specific method is as follows for ranking each individual’s sense of meaning score. For example, if an individual’s score in the “presence” stage was 5 points, whereas scores in other stages were below 5 points, the individual would be classified as being in the “presence” stage. This process was repeated for all individuals, resulting in a percentage distribution of adolescents in different stages with respect to age, as shown in Figure 1a.

Figure 1a shows the percentage of meaning in life development stages across age groups. Participants of all ages most often reported presence and search as the highest. It was unusual for adolescents to be in a state of “precontemplation”. In addition, those in an unhealthy developmental state tended to show lower levels of “foreclosure”, “diffusion”, and “ruminative exploration”. Finally, our predicted hypothesis of a progressive reduction in “precontemplation” and a corresponding increase in “presence” did not materialize, suggesting that the maturation of the sense of meaning may have been in equilibrium from an earlier period.

Second, in order to understand differences in meaning in life across age groups, the meaning in life scale was first tested for measurement invariance across age groups. The results are presented in Table 2. The results show that ΔCFI and ΔTLI were less than 0.01 in each step of the measurement equivalence test for age groups [44]. Therefore, measurement invariance tests across age groups were completely valid.

Third, multifactorial analysis of variance (MANOVA) was used to analyze the differences in meaning in life among different age groups. Moreover, Figure 1b shows significant differences in precontemplation (M = 2.21, SD = 0.90, F = 17.15, *p* < 0.001, η^2^ = 0.002), foreclosure (M = 2.92, SD = 0.84, F = 117.31, *p* < 0.001, η^2^ = 0.012), search (M = 3.83, SD = 0.78, F = 21.53, *p* < 0.001, η^2^ = 0.002), ruminative exploration (M = 2.88, SD = 0.93, F = 12.54, *p* < 0.001, η^2^ = 0.001), diffusion (M = 2.48, SD = 0.89, F = 48.58, *p* < 0.001, η^2^ = 0.005), and presence (M = 3.47, SD = 0.77, F = 75.25, *p* < 0.001, = 0.008) levels among students across different ages. Follow-up comparisons revealed that students aged 16 had significantly lower levels of foreclosure, search, and presence compared to other ages. Similarly, students aged 18 had significantly lower levels of precontemplation and diffusion compared to other ages. Finally, students aged 23 had significantly lower levels of ruminative exploration compared to other ages.

Finally, independent sample *t*-tests were used to analyze differences in meaning in life between the adolescent and emerging adult groups. Specific results are shown in Table 3. As can be seen in Table 3, there was no significant difference between the adolescent sub-group and the emerging adult sub-group for search. Precontemplation, foreclosure, ruminative exploration, diffusion, and presence were all significantly higher in the emerging adult sub-group than in the adolescent sub-group.

### 3.2. Analysis of Purpose Orientations

First, in order to better understand how youths prioritized purpose orientations, percentages of prioritization were calculated. The person-centered analysis method was used to analyze the forced choice from purpose orientations. In this approach, a forced-choice purpose orientation was assigned a higher level of importance if it was ranked first by a participant. However, if there was a tie for first place, the orientation was classified as “other”. As presented in Table 4, participants expressed 31 different distributions for purpose orientations. Family well-being was the most common purpose orientation (42.4%), followed by personal growth (20.3%), social promotion (11.8%), personal well-being (8.5%), and others (17%). It is noteworthy that the proportions of 13, 14, and 15 purpose orientations were higher than 10%.

**Figure 2:**
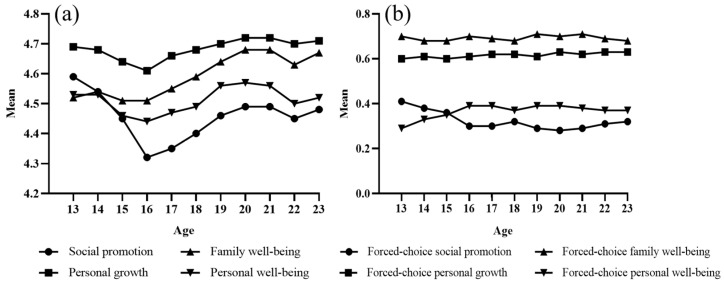
(**a**) Subscales of self-reported purpose orientations among adolescents across ages. (**b**) Subscales of forced-choice purpose orientations among adolescents across ages.

Second, we set out to understand how purpose orientations change with age. A descriptive analysis of the self-reported form and forced-choice form was conducted. The results are presented in Figure 2. Four key findings about the development of purpose orientations among young people emerged from Figure 2a,b. First, personal growth and family well-being were top priorities for youth, with personal growth being the primary focus. However, when there was a conflict between personal growth and family well-being, adolescents prioritized family well-being over personal growth. Secondly, personal growth preceded personal well-being, indicating the importance of personal growth in achieving overall well-being. Third, social promotion was the least important life purpose for youth. Fourthly, the study showed a significant decline in the pursuit of all life purposes, with a pessimistic tendency around the age of 16. However, the pursuit of all four life purposes increased significantly at the university level. The study found that personal well-being increased, whereas social promotion decreased, from age 13 to 16 before stabilizing. These findings suggest that personal growth and family well-being are important life goals for adolescents and that their priorities may shift over time.

Third, in order to understand differences in purpose orientations across age groups, the purpose orientation scale was first tested for measurement invariance across age groups. The results are presented in Table 2. The results show that ΔCFI and ΔTLI were less than 0.01 in each step of the measurement equivalence test for age groups [44]. Therefore, measurement invariance tests across age groups were completely valid.

Fourth, multifactorial analysis of variance (MANOVA) was used to analyze the differences in purpose orientations among different age groups. There were significant differences in purpose orientations between age groups. The results presented in Figure 2 show significant differences between social promotion (M = 4.46, SD = 0.66, F = 169.16, *p* < 0.001, η^2^ = 0.018), personal growth (M = 4.68, SD = 0.48, F = 40.48, *p* < 0.001, η^2^ = 0.004), family well-being (M = 4.57, SD = 0.66, F = 82.85, *p* < 0.001, η^2^ = 0.010), and personal well-being (M = 4.51, SD = 0.63, F = 42.53, *p* < 0.001, η^2^ = 0.004). Further analysis revealed that compared to other age groups, students aged 16 had significantly lower self-reported levels of social promotion, personal growth, family well-being, and personal well-being. These findings suggest significant differences in the purpose orientations of youths of different ages.

Finally, independent sample *t*-tests were used to analyze differences in purpose orientations between the adolescent and emerging adult groups. Specific results are shown in Table 3. As can be seen in Table 3, social promotion, personal growth, family well-being, and personal growth were all significantly higher in the emerging adult sub-group than in the adolescent sub-group.

### 3.3. Analysis of Attitudes toward Life

**Figure 3:**
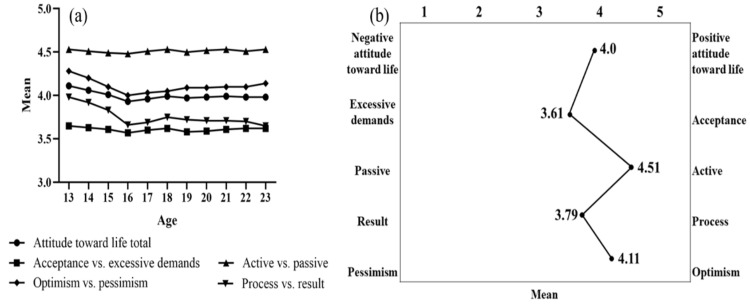
(**a**) Means of the subscales of attitudes toward life among adolescents across ages. (**b**) Means of the scales of youths’ attitudes toward life.

First, we set out to understand how attitudes toward life change with age. A descriptive analysis of attitudes toward life was conducted. The results are presented in Figure 3. As shown in Figure 3b, the higher the mean value of attitudes toward life, the more positive they were. Three notable observations can be made from Figure 3a,b. First, overall attitudes toward life were above the median. Second, young people tended to be more active and process-oriented but less optimistic than expected, and they struggled to cope with setbacks in life. Third, between the ages of 13 and 16, there was a decline in the overall attitudes toward life score, as well as in acceptance vs. excessive demands, process vs. result, and optimism vs. pessimism scores. However, after the age of 16, there was a gradual increase followed by stabilization. Notably, there was no significant decrease in active vs. passive between the ages of 13 and 16.

Second, in order to understand differences in attitudes toward life across age groups, the attitude toward life scale was first tested for measurement invariance across age groups. The results are presented in Table 2. The results show that ΔCFI and ΔTLI were less than 0.01 in each step of the measurement equivalence test for the age groups [44]. Therefore, the measurement invariance tests across age groups were completely valid.

Third, multifactorial analysis of variance (MANOVA) was used to analyze the differences in attitudes toward life among different age groups. There were significant differences in attitude toward life between age groups. Figure 3a reveals significant disparities in the attitude toward life total (M = 4.01, SD = 0.56, F = 98.39, *p* < 0.001, η^2^ = 0.01), acceptance vs. excessive demands (M = 3.61, SD = 0.66, F = 12.74, *p* < 0.001, η^2^ = 0.001), active vs. passive (M = 4.51, SD = 0.66, F = 4.97, *p* < 0.001, η^2^ = 0.001), process vs. result (M = 3.79, SD = 1.01, F = 119.48, *p* < 0.001, η^2^ = 0.013), and optimism vs. pessimism (M = 4.11, SD = 0.83, F = 102.81, *p* < 0.001, η^2^ = 0.011) levels among students across different ages. Follow-up comparisons revealed that students aged 16 had significantly lower scores than other age groups regarding their overall attitude toward life scores. Additionally, the 16-year-old age group scored significantly lower than the 13–15-year-old age group regarding the acceptance vs. excessive demands, process vs. result, and optimism vs. pessimism factors.

Finally, independent sample *t*-tests were used to analyze differences in attitudes toward life between the adolescent and emerging adult groups. Specific results are shown in Table 3. As can be seen in Table 3, there was no significant difference between the adolescent sub-group and emerging adult sub-group for active vs. passive. Attitude toward life total, acceptance vs. excessive demands, process vs. result, and optimism vs. pessimism were all significantly lower in the emerging adult sub-group than in the adolescent sub-group.

## 4. Discussion

This study surveyed 94,219 junior high, high school, and college students in 31 provinces to examine their meaning in life, purpose orientations, and attitudes toward life development across age groups. The results of the study provide important insights into the characteristics of China’s youth and could help to design educational initiatives to develop aspects related to meaning in life, purpose orientations, and attitudes toward life.

The first important finding of this study is that most youth aged 13–23 had a high level of search for meaning or the presence of meaning. The fact that most youths were in the “search for meaning” stage means, on the one hand, that most young people have already begun to explore the sense of meaning in their lives and that there is an urgent need to educate youth about the meaning in life. In contrast, a smaller percentage of youth were found to have high levels of ruminative exploration and diffusion, and the lowest percentage had high levels of precontemplation and foreclosure. Thus, the results reflect a healthy development of Chinese youths’ meaning in life. These findings are also consistent with previous research [6]. However, it is essential to analyze why adolescence is a crucial period for the initial formation of meaning in life. During this period, young people reflect on their social roles and life problems. Various influences, such as family and education, shape their thinking. Their cognitive abilities may not be fully developed, which places youth in the exploratory stage of searching for meaning in life. It is suggested that actively constructing meaning from challenging experiences may lead to better developmental outcomes for adolescents [11]. Furthermore, a large-scale survey of adolescents aged 12–15 years in seven countries found that 83% of participants had searched for meaning in life on multiple occasions, and 88% of them considered finding meaning in life to be one of the most important goals in life [45]. Therefore, the search for meaning is a crucial stage in youths’ formation of meaning in life. Furthermore, Steger argues that it is healthy for adults to have high levels of presence of meaning, whereas adults who search for meaning are more likely to experience depression and anxiety [12]. Conversely, research suggests that searching for and creating meaning during adolescence can have positive outcomes. For example, studies have shown that searching for meaning in life can reduce hopelessness, promote healthier behaviors [46], and contribute to overall well-being [11]. These findings suggest that seeking meaning in life during youth may be beneficial for personal development.

In summary, the study found that the search for meaning and the presence of meaning are two crucial phases in the formation of meaning in life. This study also suggests that it is essential to consider additional developmental stages, such as precontemplation, foreclosure, ruminative exploration, and diffusion, which have been found to influence the search for meaning in youth. Specifically, some studies have found a negative correlation between the pursuit of meaning in life in youth and well-being [47], whereas others have found a positive correlation [11]. These discrepancies may be due to the limitations of previous studies, which only considered the search for and presence of meaning in life. This study proposed precontemplation, foreclosure, ruminative exploration, and diffusion as additional developmental stages in the formation of meaning in life. Previous studies using cluster analysis methods have identified four developmental states of meaning in life: accomplished, foreclosed, uncommitted, and diffused, which supports our proposed stage classification [13,16,17].

Finally, the study did not find a gradual decrease in precontemplation or an increase in the presence of meaning with age, suggesting that the development of meaning in life may be relatively stable early on. There may be two reasons for this. On the one hand, during youth, meaning in life is usually not given much thought. On the other hand, meaning in life is formed after going through major life events. As young people between the ages of 13 and 23 do not have much experience of life, there is little formation of meaning in life during this period. This finding is also consistent with the argument that the search for meaning is a continuous process rather than a linear progression toward presence [4].

The second notable finding of the study is that personal growth and family well-being are the top priorities for youths, with personal growth being the primary focus. However, in the forced-choice scale format, youth prioritized family well-being over personal growth by a significant percentage. However, personal growth preceded personal well-being, indicating the importance of personal growth in achieving overall well-being. In addition, there was a significant decline in all purpose orientations, with a pessimistic tendency around the age of 16. The pursuit of all four purpose orientations increased significantly after the age of 16. Moreover, personal well-being increased between the ages of 13 and 16, whereas social promotion decreased and then remained stable. Therefore, these results suggest that personal growth and family well-being are crucial life purposes for youths and that their priorities may change over time.

The findings also challenge the common assumption that youths are driven by pleasure. The results of the study can be attributed to the economic development of the country and the pressure from the education sector. These findings are also consistent with the characteristics of collectivist cultures, where individuals prioritize the collective group over individual pursuits [29,32]. The results from the self-report and forced-choice forms of the purpose orientation scale showed that personal growth is a priority for youths when it is not compared to family well-being. When comparing personal growth and family well-being, youth prioritized family well-being over personal growth. Prioritizing family well-being is consistent with the traditional Chinese concept of the family as the collective and the standard for decision-making [31]. Additionally, a previous study of Taiwanese college students found similar results, with harmonious relationships with relatives and friends identified as the primary source of happiness [24]. Moreover, Maslow’s hierarchy of needs suggests belonging and love as basic needs to be satisfied before higher-level needs.

Another interesting finding is the low level of social promotion. Despite the traditional Chinese belief that youths should prioritize the interests of the country and contribute to the greater good, the findings show that youths gave the lowest priority to social promotion. Mencius stated that those who are impoverished focus on themselves, whereas those who are wealthy focus on the world [48]. Therefore, strengthening the social promotion of youths is crucial for the future and progress of the country, as they hold the key to the country’s progress.

The third important finding is that youth generally have a positive attitude toward life. This is reflected in the fact that most people believe that they should be in charge of their future rather than relying on their environment and fate, that they are responsible for their future, that they can face all possible setbacks and difficulties in life with acceptance rather than being overly stubborn, that they are more concerned with experiencing the process of life rather than the outcome, and that they can face life with an optimistic attitude. This finding is consistent with the widely held belief that youths are overly optimistic [35]. Some youths may develop a more positive attitude toward life due to physical and psychological immaturity and a lack of experience in dealing with failure and problems. Furthermore, in some situations, the safe environment provided by schools and families may lead to an overly optimistic view. This may be one of the reasons why young people have a more positive attitude toward life.

The fourth key finding of this study is that there were significant differences between age groups in young people’s meaning in life, purpose orientation, and attitudes toward life, with 16-year-olds reporting the lowest levels compared to other age groups. Nevertheless, the transition from junior high school to senior high school, with its increased academic demands, may be challenging for 16-year-olds, leading to difficulties in adjusting to academic responsibilities. This finding is consistent with previous studies that have found that students’ perceptions of their quality of life tend to deteriorate as academic expectations increase [35]. Furthermore, in the Chinese educational context, many high school teachers focus on academic performance and test scores, neglecting the development of students’ positive behaviors and character traits. This approach could increase stress levels and negative attitudes toward life among 16-year-olds as academic performance becomes their sole focus. Overall, the study emphasizes the need to create a supportive, inclusive, non-competitive learning environment that prioritizes students’ holistic development over academic achievement alone. In addition, parents need to create a home environment that fosters open communication and prioritizes the emotional and behavioral well-being of children. The development of youths’ meaning in life, purpose orientation, and life attitudes should be a priority for parents and educators to promote both academic success and overall well-being.

The final finding of this study is that emerging adults think better than adolescents about the meaning in life because precontemplation, foreclosure, ruminative exploration, diffusion, and the presence of meaning in life were all higher in emerging adults than in adolescents. This finding is also more consistent with previous research. Previous research has found that the prevalence of meaningfulness seems to increase with age [49]. Therefore, more attention should be paid to guiding adolescents to think about meaning in life issues. In addition, emerging adults know what they want better than adolescents, with all four purpose orientations—social promotion, personal growth, family well-being, and personal well-being—being significantly higher for emerging adults than for adolescents. This finding is consistent with developmental psychology. Adolescence and emerging adulthood are mature periods of developmental purpose [2,3], and as such, there may be some de-developmental growth in purpose during this period. For example, emerging adults are more likely to be purposeful than adolescents [41]. Another interesting finding is that emerging adults are not as positive about their attitude toward life as adolescents. Attitude toward life total, acceptance vs. excessive demands, process vs. result, and optimism vs. pessimism were all significantly lower for emerging adults than for adolescents. This may be related to the fact that adolescents experience fewer setbacks and hardships in life. Emerging adults, on the other hand, experience more negative life events in school and in life as they age. As a result, they do not have as positive an attitude toward life as adolescents. Although we expect emerging adults to recognize the truths of life as they age, they still love it. However, it has been found that although emerging adults recognize the truth of life, they do not love life as much and their attitude toward life becomes more negative. Therefore, how to cultivate a positive attitude toward life in emerging adulthood is an issue we should pay attention to. This is because human attitudes toward life are closely related to adolescent well-being [5].

Finding a sense of meaning in life, pursuing a self-transcendent purpose, and having a positive attitude toward life are extremely important for students’ mental health and well-being. Therefore, this study sheds light on the current status of Chinese youths’ meaning in life, purpose orientation, and attitudes toward life with a broadly representative sample. Therefore, we believe that the findings will make an important academic and practical contribution to the developing field of positive development, including adolescent mental health and well-being. The search for meaning in life, purpose orientation, positive attitudes toward life, and how this construct contributes to optimal human development is a topic of increasing interest to positive psychology scholars, and the results of this study will reveal at what age meaning in life, purpose orientation, and life attitudes serve as indicators of and contributors to student well-being. In addition, the results will shed light on the nature of life attitudes, a more externality-oriented concept that has become increasingly relevant to positive youth development researchers. Specifically, the results of the study will contribute to meaning in life, purpose orientations, and attitudes toward life. From a practical perspective, the findings will provide information on how and when to effectively develop meaning in life, purpose orientations, and attitudes toward life. Specifically, the findings will reveal what youths pursue, what constitutes a life worth living for youths, and what youths’ attitudes toward life look like, and give educators direction for finding meaning in life, purpose orientation, and positive attitudes toward life for students. It is also an important role for parents and teachers to guide students in exploring a sense of meaning in life, establishing a sense of purpose, and increasing their satisfaction with their attitudes toward life. On the one hand, we encourage parents to provide more support and encouragement for their children to enhance their exploration of meaning and purpose and further develop a positive attitude toward life. On the other hand, we suggest that schools create an environment to provide more life education programs and activities to enhance students’ self-satisfaction with their own performance so that they can learn the importance of appreciating and enjoying life. This will further improve the mental health and well-being of students. At the same time, it is widely recognized that students need a warm, supportive, and friendly environment to realize their full potential. In particular, students at the age of 16 need more encouragement and care in their emotions and studies so that they are better able to finding a sense of meaning in life, discover a sense of purposeful orientation, and possess the courage to face setbacks, that is, have a positive attitude toward life. This will help students have a healthy and happy life. This will have a significant impact on fostering and supporting the development of adolescents’ and emerging adults’ meaning in life, purpose orientations, and attitudes toward life, which in turn will have a significant impact on students’ mental health and well-being.

### Limitations and Future Directions

This study has several limitations. First, despite the relatively large sample size, convenience sampling is a double-edged sword. Although it increases the generalizability of the study, it may also introduce selection bias. In addition, cross-sectional research designs may not fully capture how adolescents’ meaning, purpose, and life attitudes change over time. Future research could use longitudinal or retrospective comparisons to compare the same students’ meaning in life, purpose orientations, and attitudes toward life. Second, a mixed-methods research design would allow us to examine intra-individual change and provide a mix of qualitative, quantitative, and observational data. This will provide hard evidence that actually relates to changes in adolescent development and allow for potential interpretations of the findings [50]. Third, the questionnaire used in this study was developed in the cultural context of China but is somewhat culturally specific due to the content of the purpose. It would be interesting for future research to examine the same meaning in life, purpose orientations, and attitudes toward life in different countries—an expanded global data set would support the robustness and validity of the methodology. Fourth, the effect sizes of the tests of variance in this study were small, and future research could use stratified sampling to confirm the consistency of the variances. Finally, further prospective research could be conducted on the factors that influence adolescents’ meaning, purpose orientations, and attitudes toward life, which may help identify more effective methods and strategies for positively cultivating these concepts in adolescents.

## 5. Conclusions

The purpose of this study was to examine the meaning in life, purpose orientations, and attitudes toward life of youths in 31 provinces in China. The results indicate that search and presence are the primary developmental stages in the formation of meaning in life. Furthermore, the pursuit of family well-being and personal growth emerged as more desirable than the pursuit of social promotion and personal well-being. In terms of attitudes toward life, youths are generally positive. In addition, the age of 16 is a crucial turning point in the development of youths’ meaning in life, purpose orientations, and attitudes toward life. Emerging adults have a deeper presence for meaning in life and a better idea of what they want than adolescents. Yet, emerging adults have a lower positive attitude toward life than adolescents. Therefore, we need to pay attention to cultivating meaning in life and purpose orientation among adolescents and pay attention to the attitudes toward life of emerging adults, thus promoting the well-being of youths.

## Figures and Tables

**Table 1:** Sample distribution.

Age	Gender		N	Percentage (%)
	Male	Female		
13	6026	6683	12,709	13.5
14	7323	7447	14,770	15.6
15	3866	3626	7492	8.0
16	3963	5619	9582	10.2
17	5794	7639	13,433	14.3
18	2740	3986	6726	7.1
19	3582	5803	9385	10.0
20	3470	6586	10,056	10.6
21	2138	3735	5873	6.2
22	1056	1275	2331	2.5
23	925	937	1862	2.0
Total	40,883	53,336	94,219	100

**Table 2:** Measurement invariance of meaning in life, purpose orientations, and attitudes toward life across ages.

Model	χ^2^	df	CFI	TLI	RMSEA (90%CI)	SRMR	Model Comparison	ΔCFI	ΔTLI
Meaning in life									
Model 1	100,791.91	3124	0.925	0.908	0.060 [0.060, 0.061]	0.053			
Model 2	101,367.29	3324	0.924	0.916	0.059 [0.058, 0.059]	0.053	2 vs. 1	−0.001	0.008
Model 3	103,921.51	3524	0.922	0.921	0.058 [0.057, 0.058]	0.053	3 vs. 2	−0.002	0.005
Model 4	108,558.35	3784	0.914	0.915	0.065 [0.065, 0.066]	0.061	4 vs. 3	−0.008	−0.006
Purpose orientations									
Model 1	90,020.56	1606	0.923	0.908	0.070 [0.070, 0.071]	0.049			
Model 2	92,382.52	1756	0.920	0.914	0.068 [0.067, 0.068]	0.057	2 vs. 1	−0.003	0.006
Model 3	96,055.53	1906	0.917	0.918	0.066 [0.066, 0.067]	0.060	3 vs. 2	−0.003	0.004
Model 4	98,310.92	2056	0.913	0.911	0.071 [0.070, 0.071]	0.061	4 vs. 3	−0.004	−0.007
Attitudes toward life									
Model 1	33,391.94	528	0.928	0.895	0.075 [0.074, 0.076]	0.051			
Model 2	33,811.33	608	0.927	0.899	0.070 [0.069, 0.071]	0.053	2 vs. 1	−0.001	0.004
Model 3	36,942.74	688	0.923	0.905	0.068 [0.068, 0.069]	0.057	3 vs. 2	−0.004	0.006
Model 4	38,808.06	808	0.916	0.911	0.064 [0.063, 0.065]	0.061	4 vs. 3	−0.007	0.006

Note: Model 1 = Configural Invariance; Model 2 = Metric Invariance; Model 3 = Scalar Invariance; Model 4 = Error Variance Invariance.

**Table 3:** The *t*-test for meaning in life, purpose orientations, and attitudes toward life across adolescent sub-groups and emerging adult sub-groups.

	Adolescent		Emerging Adult				
	M	SD	M	SD	t	*p*	Cohen’s d
Meaning in Life							
Precontemplation	2.19	0.91	2.25	0.88	−9.40	<0.001	−0.07
Foreclosure	2.88	0.87	3.00	0.75	−20.34	<0.001	−0.14
Search	3.84	0.81	3.82	0.72	2.81	0.005	0.02
Ruminative exploration	2.85	0.95	2.88	0.89	−7.08	<0.001	0.05
Diffusion	2.44	0.90	2.57	0.87	−20.02	<0.001	−0.14
Presence	3.46	0.79	3.51	0.71	−9.26	<0.001	−0.07
Purpose Orientations							
Social promotion	4.45	0.66	4.48	0.64	−4.71	<0.001	−0.03
Personal growth	4.66	0.49	4.71	0.46	−14.26	<0.001	−0.10
Family well-being	4.54	0.69	4.66	0.59	−26.79	<0.001	−0.19
Personal well-being	4.49	0.64	4.56	0.60	−14.62	<0.001	−0.10
Attitudes toward Life							
Attitude toward life total	4.02	0.56	3.98	0.55	10.19	<0.001	0.07
Acceptance (vs. excessive demands)	3.62	0.67	3.59	0.65	5.08	<0.001	0.04
Active (vs. passive)	4.51	0.67	4.51	0.65	−0.74	0.457	−0.01
Process (vs. result)	3.82	1.02	3.71	1.00	15.36	<0.001	0.11
Optimism (vs. pessimism)	4.12	0.84	4.09	0.81	5.18	<0.001	0.04

**Table 4:** The 31 distributions of forced-choice purpose orientations among youths.

	Percentage (%)	Permutations and Combinations of Four Purpose Orientations	Percentage (%)
Social promotion	11.8	(1) SP, PG, PW, FW	3.4
		(2) SP, PG, FW, PW	4.0
		(3) SP, PW, PG, FW	0.3
		(4) SP, PW, FW, PG	0.2
		(5) SP, FW, PG, PW	3.4
		(6) SP, FW, PW, PG	0.5
Personal growth	20.3	(7) PG, PW, FW, SP	2.4
		(8) PG, PW, SP, FW	1.3
		(9) PG, FW, PW, SP	4.8
		(10) PG, FW, SP, PW	6.1
		(11) PG, SP, PW, FW	1.2
		(12) PG, SP, FW, PW	4.5
Family well-being	42.4	(13) FW, PG, PW, SP	10.5
		(14) FW, PG, SP, PW	12.5
		(15) FW, PW, PG, SP	11.0
		(16) FW, PW, SP, PG	1.1
		(17) FW, SP, PG, PW	3.9
		(18) FW, SP, PW, PG	3.4
Personal well-being	8.5	(19) PW, PG, FW, SP	2.9
		(20) PW, PG, SP, FW	1.9
		(21) PW, FW, PG, SP	2.7
		(22) PW, FW, SP, PG	0.4
		(23) PW, SP, PG, FW	0.4
		(24) PW, SP, FW, PG	0.2
Others	17	(25) PG = FW, SP = PW	3.9
		(26) PG = FW = PW, SP	3.7
		(27) SP = PG = FW, PW	2.8
		(28) FW = PW, SP = PG	2.4
		(29) PG = PW, SP = FW	1.9
		(30) SP = PG, FW = PW	1.8
		(31) SP = PG = PW, FW	0.5

Note: SP = Social Promotion; PG = Personal Growth; PW = Personal Well-being; FW = Family Well-being.

## Data Availability

The data that supports the findings of this study are contained within the article and available from the corresponding author upon reasonable request.
